# Phenotypic and genotypic analysis of antimicrobial resistance and population structure of gastroenteritis-related *Aeromonas* isolates

**DOI:** 10.1186/s12941-024-00706-2

**Published:** 2024-05-23

**Authors:** Dana Sagas, Yizhak Hershko, Katia Levitskyi, Merav Strauss, Matan Slutzkin, Bibiana Chazan, Amos Adler

**Affiliations:** 1 Clinical Microbiology, Emek Medical Center, Clalit Health Services, Afula, Israel; 2 Department of Epidemiology and Preventative Medicine, School of Public Health, Faculty of Medicine, Tel Aviv University, Tel Aviv, Israel; 3 Clinical Microbiology, Tel Aviv Sourasky Medical Center, 6 Weizmann Street, Tel Aviv, Israel; 4 Infectious Diseases Unit, Emek Medical Center, Clalit Health Services, Afula, Israel; 5 Rappaport Faculty of Medicine, The Technion-Israel Institute of Technology, Haifa, Israel

**Keywords:** *Aeromonas*, Gastroenteritis, Antimicrobial resistance, Whole genome sequencing

## Abstract

**Background:**

The population structure and the correlation between antimicrobial resistance (AMR) phenotypes and genotypes in *Aeromonas* species isolated from patients with gastroenteritis are not well understood. The aims of the study were to: (1) investigate the antimicrobial susceptibility profiles of *Aeromonas* species isolated from patients with gastroenteritis; (2) explore the relationship between AMR genes and resistance phenotypes; and (3) describe the population structure of these isolates and provide evidence of transmission events among them.

**Methods:**

This microbiological survey was performed at the Microbiology Laboratory of the Emek Medical Center in Afula, Israel. Cultivation of *Aeromonas* was attempted from stool samples that tested positive by PCR. Antimicrobial susceptibility testing (AST) was performed using the Sensititre GN3F microdilution panel. Whole genome sequencing (WGS) was done using the Illumina NextSeq500/550 system. Phylogenetic studies involved multi-locus sequence typing (MLST) and core genome (cg) MLST. Resistance mechanisms were identified using the Comprehensive Antibiotic Resistance Database and compared with the AST results.

**Results:**

The study included 67 patient-unique isolates. The species that were identified included *A. caviae* (*n* = 58), *A. dhakensis* (*n* = 3), *A. media* (*n* = 2), *A. veronii* (*n* = 2) and *A. hydrophila* (*n* = 2). Isolates were almost uniformly susceptible to amikacin, gentamicin, aztreonam, cefepime, ceftazidime, ciprofloxacin and meropenem. All isolates with the exception of 1–2 isolates were resistant to ampicillin, cefazolin and ampicillin-sulbactam which was compatible with the presence of the *bla*_OXA_ genes. Variable resistance rates were observed to cefuroxime, cefoxitin, ceftriaxone, piperacillin-tazobactam that were not correlated with the presence of other β-lactamase genes. Resistance to tetracycline and trimethoprim-sulfamethoxazole correlated with the presence of *tetA* and *sul1*, respectively. The population structure of *A. caviae* was highly diverse with the minority of the isolates (16/57) clustering into six defined sequence types. A cgMLST-based distance of four genes was found in one pair of isolates, suggesting common source transmission.

**Conclusions:**

*A. caviae* is the dominant species related to gastroenteritis and is characterized by a diverse population structure, with almost no evidence for common-source transmission. Resistance rates to most antimicrobial agents were low and partially matched with the presence of resistance genes.

**Supplementary Information:**

The online version contains supplementary material available at 10.1186/s12941-024-00706-2.

## Introduction

*Aeromonas* species are capable of causing a variety of different infections, including gastro-intestinal and extraintestinal infections [1]. Defining the precise role of *Aeromonas* species in causing gastroenteritis (GE) proves challenging, particularly outside of isolated outbreaks. This challenge arises from the variability in clinical symptoms, the absence of a specific clinical profile, the presence of *Aeromonas* in asymptomatic individuals [2] and by the frequent isolation of other potential pathogens in the same stool sample [3]. Consequently, *Aeromonas* species are not consistently included in routine stool sample testing [4] and the available data on these infections remain relatively limited.

A previous study reported the rate of antimicrobial resistance (AMR) in *Aeromonas* isolated from stool samples but did not conduct an analysis of resistance mechanisms [4]. A recent study of *Aeromonas* isolated mainly from hepato-biliary infections did not find a correlation between AMR phenotype and resistance genes [5]. Furthermore, as *Aeromonas* gastroenteritis is a food-borne infection, understanding the population structure of these infections could aid in deciphering transmission dynamics within the community.

Our laboratory, serving both the hospital and the surrounding community, commenced testing for *Aeromonas* gastroenteritis in December 2021, providing us with an opportunity to address these questions. Thus, the study aimed to: (1) investigate the antimicrobial susceptibility profiles of *Aeromonas* species isolated from patients with gastroenteritis; (2) explore the relationship between AMR genes and resistance phenotypes; and (3) describe the population structure of these isolates and provide evidence of transmission events among them.

## Methods

### Setup and population

This microbiological survey study was performed at the Microbiology Laboratory of the Emek Medical Center (EMC) in Afula, Israel. The EMC laboratory serves as the regional laboratory for a population of about 0.5 million of Clalit Health Services members, living predominantly in rural settlements and small urban centers.

Whole-genome sequencing and bioinformatics analysis were performed at the Tel Aviv Sourasky Medical Center Microbiology laboratory.

### Study design

The study was part of a prospective survey of bacterial gastroenteritis conducted between December 2021 until October 2022 [6]. All stool samples submitted to the EMC laboratory underwent PCR testing for *Aeromonas* according to the routine laboratory protocol. Samples that tested positive for *Aeromonas* by PCR were then cultured (details below), and one *Aeromonas* isolate per patient was included in the study.

The study was approved by the Ethical Review Board of the EMC.

### Identification and cultivation of Aeromonas from stool samples

Stool samples were transported from the community clinics and were tested daily except on weekends, where part of each sample was suspended in ASL buffer (Qiagen, Hilden, Germany) and refrigerated at 4 °C until tested by PCR only (culturing was done from the original sample tube). Multiplex PCR for *Aeromonas* and other bacterial pathogens was performed as previously described [7]. Stool samples that were positive by PCR for *Aeromonas* were inoculated into alkaline peptone water 0.5 M NaCl with Cephalothin (10 mg/l) and incubated overnight [8] followed by sub-culturing onto SS agar plates (Hylabs, Rehovot, Israel). Presumptive *Aeromonas* colonies were identified by MALDI Biotyper Sirius system (Bruker Daltonics, Bremen, Germany) using the MBT IVD Library Revision software. Definite determination of species was based on whole genome sequencing as described below.

### Antimicrobial susceptibility testing (AST)

AST were performed at the EMC laboratory using the Sensititre GN3F microdilution panel (Thermo Fisher Scientific) following the manufacturer’s instructions. AST included amikacin, ampicillin, ampicillin-sulbactam, aztreonam, cefazolin, cefepime, cefoxitin, ceftazidime, ceftriaxone, cefuroxime, ciprofloxacin, gentamicin, meropenem, piperacillin-tazobactam, tetracycline, tobramycin and trimethoprim-sulfamethoxazole (SXT). AST breakpoints were interpreted (when available) in accordance with the CLSI recommendations for *Aeromonas* species [9] or if absent (including ampicillin-sulbactam, aztreonam, cefazolin, cefepime, cefoxitin, cefuroxime and tobramycin), in accordance with the recommendations to *Enterobacterales* [10].

### Whole genome sequencing and bioinformatic analyses

Whole genome sequencing (WGS) was done using the Illumina NextSeq500/550 system. Libraries were prepared using Illumina DNA Prep (Illumina,20,060,059). The IDT for Illumina DNA/RNA UD Indexes (Illumina, 20,027,213) were used to tagment the DNA libraries for sequencing. After sequencing of each library, FASTQ files were imported into CLC Genomics Workbench version 23.0.5(Qiagen, Denmark). Following sequencing, the reads underwent quality trimming and contigs were assembled using the CLC Genomics Workbench version 23 (Qiagen, Denmark), with the following sequences applied as a template: *A. caviae* NZ_AP022254, *A. hydrophila* CP000426, *A. media* CP118939, *A. dhakensis* CP000462 and *A. veronii* NZ_LKKE01000001-NZ_LKKD01000048. Identification of resistance mechanisms was conducted through a combined approach, which involved (i) annotating and identifying known acquired antibiotic-resistant genes using the Comprehensive Antibiotic Resistance Database (CARD) [11], with a cutoffs of 60% identity and 80% coverage; (ii) detection of nonsynonymous mutations of selected genes and examining their correlation with resistance traits using a multi-sequence alignment approach by AliView version 1.27. Identification of protein domain sites was done using the InterProScan software [12], in comparison with *A. caviae* WP_063864115. Species designation was based on the reported average nucleotide identity. This method involved comparing the genome sequence of the tested isolates with those of reference type strains accessible in the GenBank database [13].

### Core genome (cg) multi locus sequence typing (MLST) scheme and phylogenetic analyses

We employed the chewBBACA [14] to develop two distinct whole-genome (wg) sequencing schemas, later refined into core genome (cg) schemas. The first schema encompassed 67 isolates representing five different *Aeromonas* species, and the second was exclusively tailored to the 58 isolates of *A. caviae*. Prodigal [15] (version 2.6.3) facilitated the identification of Coding DNA Sequences (CDS) for both schemas, while BLAST+ [16] (version 2.9.0) was used for conducting allelic comparisons. The initial phase involved constructing a wgMLST schema that included all CDS from the isolates, followed by the removal of paralogous alleles to establish the cgMLST schema. During the “CreateSchema” phase, each genome was annotated for pairwise comparisons, leading to an extensive all-against-all BLASTP search. The resulting BLAST score ratio (BSR) was calculated, with genes encoding identical or nearly identical proteins (BSR exceeding 0.6 by default) consolidated into a single database, representing alleles of the same locus.

For the *A. caviae*-specific schema, a cross-reference with the sequence types from PubMLST was also performed to enhance the contextual understanding of the isolates. The allelic profiles derived from the chewBBACA cgMLST schemas of both groups were then subjected to phylogenetic analysis using GrapeTree software (version 1.5.0). Neighbor Joining (NJ) and Minimum Spanning Trees were constructed for each schema, with the trees generated from the allelic profiles being visualized in iTOL [17]. This comprehensive approach allowed for a detailed exploration of the genetic relationships within and between the diverse *Aeromonas* spp., with a specific focus on the *A. caviae* isolates. Two isolates derived from two separate samples of the same patient were included in the analysis. As these two isolates were assumed to be directly related, they were included in order to provide an epidemiological control for the phylogenetic schema, i.e., the number of gene-differences that can be expected to reflect direct epidemiological linkage.

### Data availability

This Whole Genome Shotgun project has been deposited in NCBI under BioProject accession number PRJNA1040111. In addition, all 58 *A. caviae* isolates were deposited in PubMLST under ID BIGSdb_20231207071129_2157505_94891.

### Statistical analysis

MIC_50_ and MIC_90_ were calculated using RStudio software, version 4.1.2. Graphs were drawn using the ggplot2 package in R.

## Results

### Antimicrobial susceptibility profiles of Aeromonas species

**Fig. 1:**
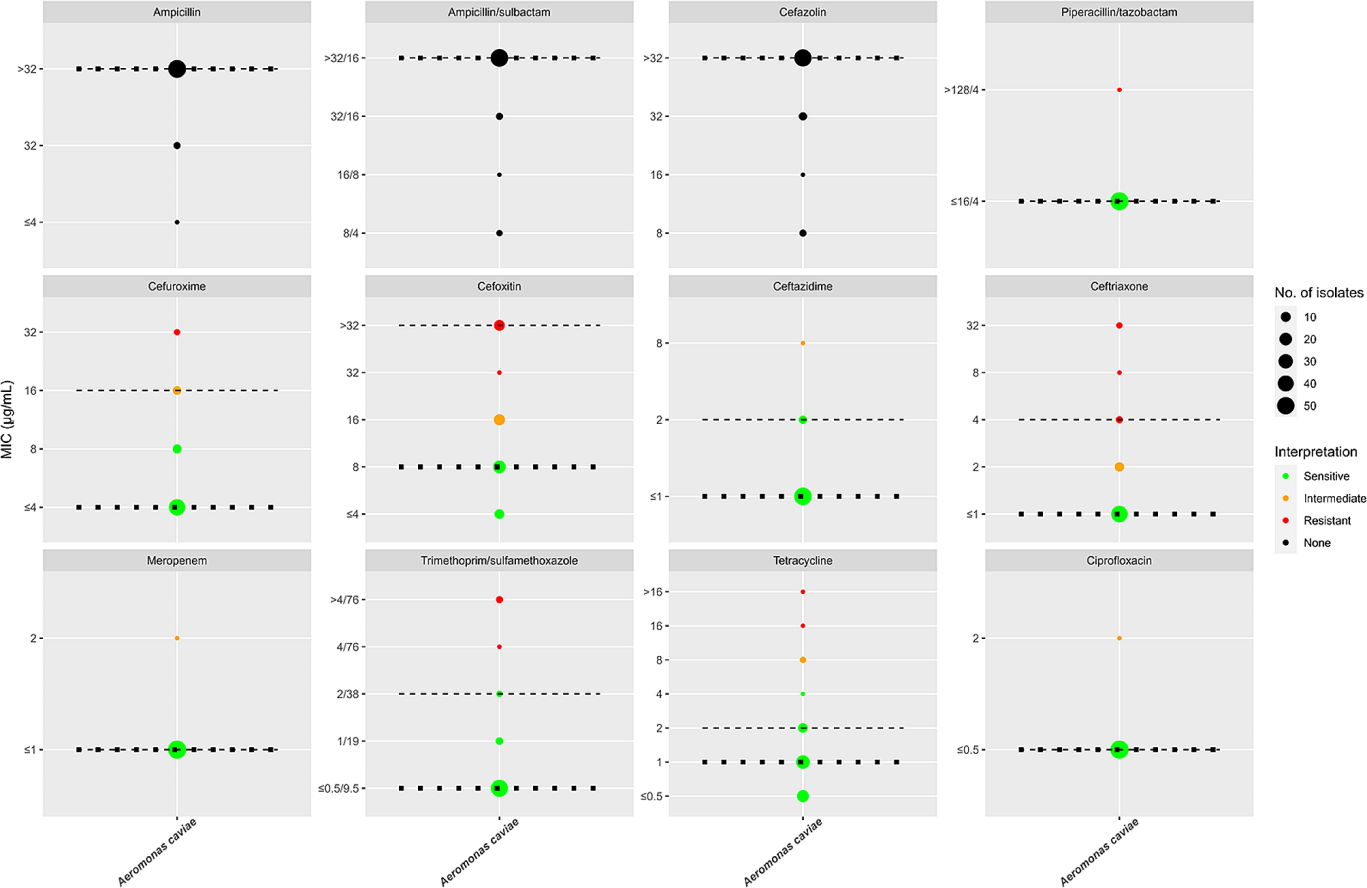
The MIC distributions of 12 antimicrobials for 58 *A. caviae* isolates. The red, orange and green dots indicate the resistant, intermediate and susceptible breakpoints, respectively. Black dots were used if no breakpoints were available. MIC_50_ and MIC_90_ values are indicated by black dotted and black dashed lines, respectively. Dot/dash lines indicate identical MIC_50_ and MIC_90_ values

During the study period, *Aeromonas* species were isolated from 67 patient-unique stool samples and underwent WGS. The distribution of species was as follows: *A. caviae* (*n* = 58), *A. dhakensis* (*n* = 3), *A. madia* (*n* = 2), *A. veronii* (*n* = 2) and *A. hydrophila* (*n* = 2). Compared with WGS, MALDI-ToF correctly identified 52 of the 67 isolates (77.6%) with all *A. dhakensis* isolates misidentified as *A. hydrophila*. A second *A. caviae* isolate from an additional sample from the same patient was employed as a control for the phylogenetic analysis (see below). The results of the AST of the 58 *A. caviae* isolates are presented in Fig. 1 and table S1. *A. caviae* isolates were uniformly susceptible to amikacin, gentamicin, aztreonam and cefepime. Also, with the exception of 1–2 isolates, all were susceptible to ceftazidime, ciprofloxacin and meropenem. In contrast, all isolates with the exception of 1–2 isolates were resistant to ampicillin, cefazolin and ampicillin-sulbactam. A more variable distribution of the MIC values was observed with the β-lactam antimicrobials cefuroxime, cefoxitin, ceftriaxone and piperacillin-tazobactam and with tetracycline and SXT.

Similar patterns of antimicrobials susceptibility profiles were observed with the non-caviae *Aeromonas* species (table S1).

### Genotypic analysis of antimicrobial resistance in Aeromonas species

**Fig. 2:**
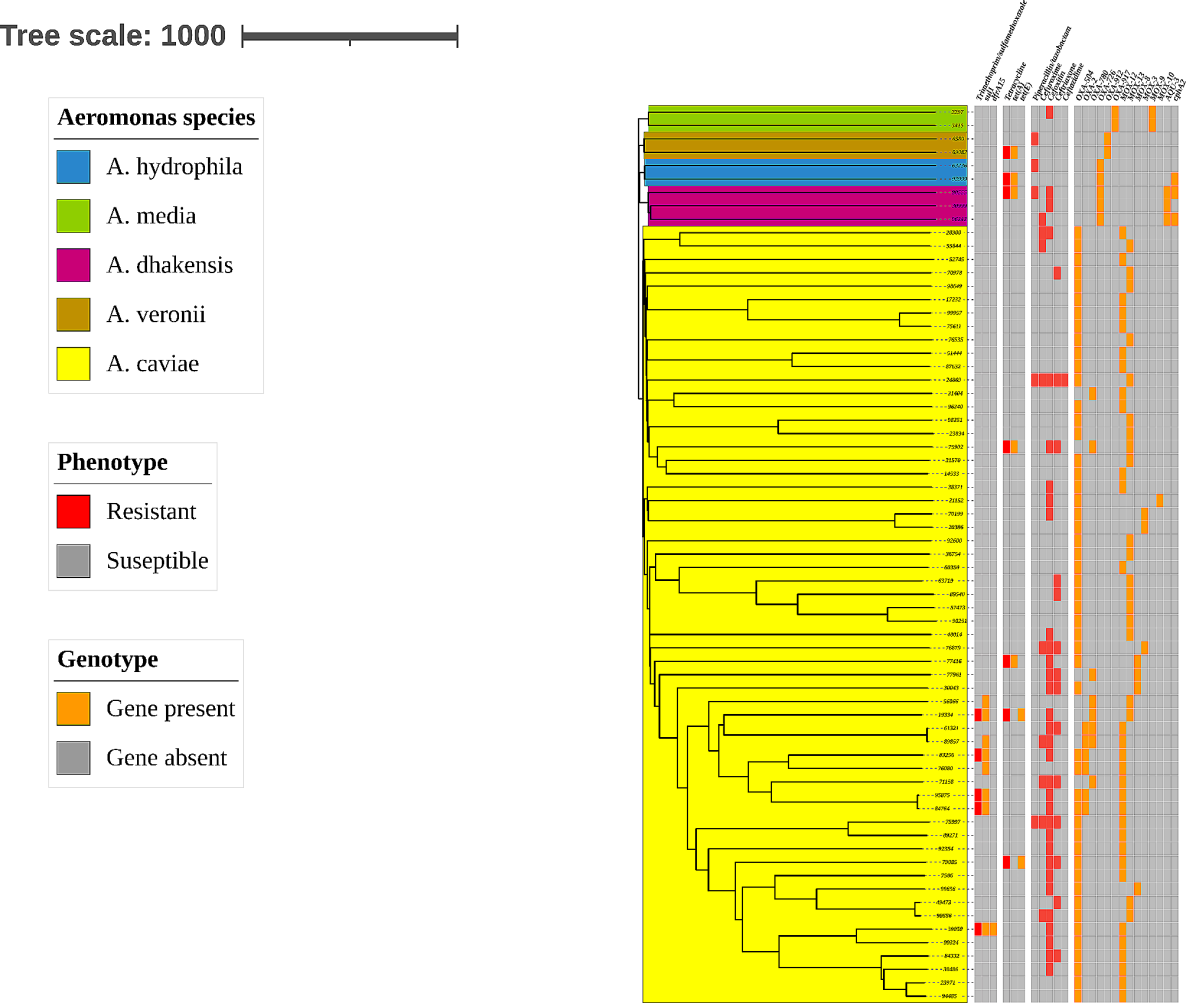
Antimicrobial phenotypic-genotypic correlations of *Aeromonas* species. The correlation is presented in the context of a cgMLST-based neighbor-joining tree analysis. The phenotypic-genotypic correlations are presented from left to right for SXT, tetracycline and β-lactam antimicrobials, respectively

Tetracycline and SXT: The phenotypic-molecular correlations for tetracycline and SXT resistance are presented in Fig. 2a. Resistance to tetracycline was related with the presence of the *tet(A)* gene, whereas SXT resistance was related to the presence of the *sul1* gene. Two susceptible isolate also possessed the *sul1* gene but had a relatively elevated MIC compared with the other susceptible isolates (2/38 vs. ≤0.5/9.5, respectively).

β-lactam antimicrobials: The phenotypic-molecular correlations for cefuroxime, cefoxitin, ceftriaxone, ceftazidime and piperacillin-tazobactam resistance are presented in Fig. 2b. All *A. caviae* isolates harbored at least one *bla*_OXA_ and *bla*_MOX_ types genes, with the most common alleles being *bla*_OXA−504_, *bla*_MOX−12_ and *bla*_MOX−13_. Six isolates also harbored a second *bla*_OXA_ gene allele. No correlation was observed between the allele type or the presence of more than one *bla*_OXA_ gene allele and the presence of resistance to the β-lactam antimicrobials tested. Other *bla*_OXA_ alleles alone or in addition to the *bla*_OXA−504_ gene were present in a 11-isolate phylogenetic cluster.

As mentioned above, almost all isolates were resistant to ampicillin, cefazolin and ampicillin-sulbactam which was compatible with the presence of the *bla*_OXA_ genes. The single *A. caviae* isolate that was susceptible to all of these agents harbored both *bla*_OXA−504_ and *bla*_MOX−13_. Comparative analysis of the *bla*_OXA−504_ gene protein sequence identified a S53Y substitution at the enzyme active site.

In species other than *A. caviae*, all isolates harbored one *bla*_OXA_ gene of different alleles than *A. caviae* but only two isolates harbored *bla*_MOX−9_. Other β-lactamase that were detected included *bla*_cphA_, *bla*_ceps_ and *bla*_AQU_. As with *A. caviae*, the presence of the *bla*_OXA_ gene correlated with the resistance to ampicillin, cefazolin and ampicillin-sulbactam but no correlation was found with the resistance phenotypes to the other β-lactam antimicrobials.

### Phylogenetic analysis of Aeromonas caviae isolates

**Fig. 3:**
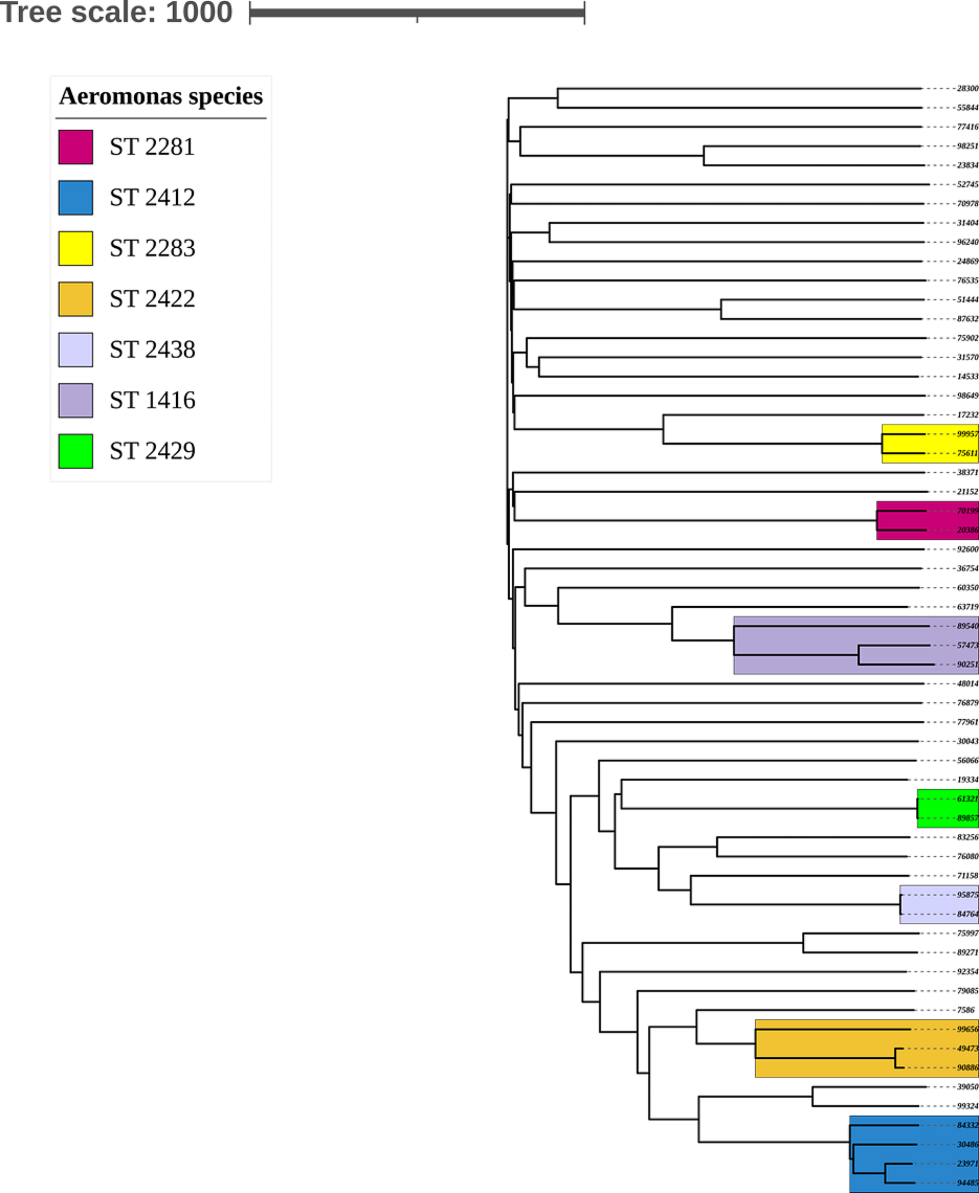
CgMLST-based neighbor-joining tree comprising 58 *A. caviae* isolates. Sequence types (ST) are marked in different colors according to the nomenclature of the 6-loci MLST scheme hosted on PubMLST

The cgMLST-based phylogenetic analysis of *A. caviae* isolates is presented in Fig. 3 and S1. The population structure was highly diverse with the minority of the isolates (16/57) clustering into six defined sequence types (ST) (Fig. 3 and S2). Isolates 95,875 and 84,764 were obtained from the same patient and were included in the analysis as control. Both of them were identified as ST-2438 with only 7-gene difference in cgMLST analysis (figure S1). The numbers of cgMLST gene difference between isolates within the same ST were higher in most ST clusters with the exception of two ST-2429 isolates (from two patients), were the cgMLST gene difference was 4 genes.

## Discussion

Our study of the molecular features of gastroenteritis-related *Aeromonas* species was initiated following the transition from culture to PCR-based diagnosis of bacterial gastroenteritis [6]. Prior to this transition, *Aeromonas* was not routinely tested in stool cultures in our laboratory (or anywhere in Israel) and thus the species distribution in our community was unknown. The predominant species in our study was *A. caviae* (58/67), with four other species accounting for the rest of the isolates. *A. caviae* was also reported as the predominant species (69%) in a previous study of *Aeromonas* gastroenteritis North-Western Israel [4], followed by *A. veronii* in 29%. Globally, four species account for most cases of gastroenteritis: *A. hydrophila*, *A. caviae*, *A. veronii biovar.sobria*, and *A. trota*; *A. caviae* were the predominant species in most of the reviewed studies [2] We also identified three cases of *A. dhakensis*, a species that is rarely isolated from stool culture and is typically found in tropical areas [18].

AST results for the dominant species (*A. caviae*) showed relatively low variability, with the vast majority of isolates being either susceptible (e.g., ceftazidime, ciprofloxacin) or resistant to the tested antimicrobial drugs (e.g., ampicillin, cefazolin). More variability was found in several antimicrobial drugs, such as cefuroxime, ceftriaxone, tetracycline and SXT. Several previous studies [4, 5, 19] have demonstrated similar variability in susceptibility profiles, while others [19] have indicated nearly complete susceptibility. As we move into the current era, the diagnosis of bacterial gastroenteritis is increasingly shifting towards PCR rather than culture-based detection [6]. Consequently, *Aeromonas* gastroenteritis is also expected to be diagnosed more frequently through PCR in the future. Regretfully, AST reports are less likely to be available for the clinician [20] and the selection of antimicrobial treatment might be guided mostly based on pre-existing data. The cumulative data from our study and previous study suggests that among the oral agents, fluoroquinolones are probably the most appropriate choice [1].

Our study was aimed to explore the molecular mechanism beyond AMR in *Aeromonas* gastroenteritis-related isolates. Here, our goal was only partially achieved as we were able to provide an explanation for the uncommon resistance phenotypes to SXT and tetracycline (*sul1* and *tetA/E*, respectively) as previously described [21, 22]. The picture regarding the β-lactam antimicrobials was more complex. All isolates harbored a *bla*_OXA_ type genes, all *A. caviae* harbored a *bla*_MOX_ type genes, while non-*caviae Aeromonas* species harbored additional types of β-lactamase. These genetic profiles matched with the resistance to ampicillin, cefazolin and ampicillin-sulbactam, as reported by some but not all studies [23]. However, we could not find an explanation for the few cases of ampicillin or cefazolin susceptibility with the exception of one case with a S53Y substitution in the *bla*_OXA−504_ gene protein sequence which could have possibly altered the activity of this enzyme. The S53Y substitution in the *bla*_OXA−504_ gene protein has never been reported before, thus necessitating validation of its biochemical effect in-vitro. We also could not identify a correlation between the genomic features and the resistance to 2nd - or 3rd -generation cephalosporin phenotypes. This lack of correlation was also reported by a previous study [5] and is likely the result of de-repression of the class C β-lactamase expression [24]. Thus, genomic studies are probably limited in their ability to decipher β-lactamase resistance in *Aeromonas* species which requires also a gene expression analysis.

Using the standard MLST schema as reference, we found that the majority of isolates did not cluster into specific ST. This is similar to the results of *Aeromonas* species isolates from various clinical sites [5]. In a study of *A. veronii* isolated from patients with gastroenteritis the isolates were defined as “closely related” [25]; However, given the differences in WGS methodology and the absence of MLST as a reference, evaluating this description in comparison with our results is challenging. Furthermore, through the use of cgMLST, we were able to illustrate that apart from one pair of isolates, the differences between isolates, even within the same ST, were considerable. This finding suggests that direct or common-source transmission is unlikely.

In addition to the previously mentioned limitations concerning the correlations between AMR phenotypes and genotypes, our study’s ability to comprehensively represent the bacterial population of *Aeromonas* gastroenteritis-related isolates may have been constrained. This limitation stemmed from culturing being performed only following a positive PCR result, and it was not always successful (merely 68 out of 283 PCR-positive cases, 24%). Additional possible cause for the low rate of culture positivity might have been less than optimal choice of the selective/enrichment media. A low rate of culture positivity versus PCR (0.34 versus 2.9, 8.5%) was also noted in a before-after study of the same PCR kit [7]. Consequently, we were unable to include all cases within the defined period, potentially hindering the identification of putative transmission chains. Despite these limitations, our study offers additional insights into the prevalence and mechanisms of AMR, as well as novel data regarding the population structure of *Aeromonas* gastroenteritis-related isolates.

### Electronic supplementary material

Below is the link to the electronic supplementary material.

Supplementary Material 1

## Data Availability

Availability of data and materials: The NGS datasets generated during the current study are available under BioProject accession number PRJNA1040111. In addition, all 58 A. caviae isolates were deposited in PubMLST under ID BIGSdb_20231207071129_2157505_94891.
