# Supplementary material for: Phenotypic and genotypic analysis of antimicrobial resistance and population structure of gastroenteritis-related Aeromonas isolates
Source: Ann Clin Microbiol Antimicrob. 2024 May 23;23:45. doi: 10.1186/s12941-024-00706-2 (PMC11119697; doi:10.1186/s12941-024-00706-2)
Supplement: Supplementary file 1 — Supplementary Material 1 [file 12941_2024_706_MOESM1_ESM.docx]

|  |  |  | Susceptible/Intermediate/Resistant (%),[MIC_50_:MIC_90_] | | | | | | | | | | | | | | | | |
| --- | --- | --- | --- | --- | --- | --- | --- | --- | --- | --- | --- | --- | --- | --- | --- | --- | --- | --- | --- |
| **Species** | **No. of isolates** | **AMK** | **GEN** | **TOB** | **TG** | **ATM** | **CIP** | **SXT** | **TET** | **MER** | **AMP** | **AMP/SUL** | **PIP/TAZ** | **CFZ** | **CAZ** | **AXO** | **FEP** | **CXM** | **FOX** |
| 1. *caviae* | 58 | 100/0/0 | 100/0/0 | [≤4: ≤4] | [≤1: ≤1] | [≤4: ≤4] | 100/0/0 | 91/0/9 | 93/3/5 | 98/0/2 | [>32: >32] | [>32/16: >32/16] | 96/2/2 | [>32: >32] | 98/2/0 | 76/14/10 | [≤4: ≤4] | [≤4: 16] | [8: >32] |
| 1. *veronii* | 2 | 100/0/0 | 100/0/0 | [≤4: ≤4] | [≤1: ≤1] | [≤4: ≤4] | 100/0/0 | 100/0/0 | 50/0/50 | 100/0/0 | [>32: >32] | [>32/16: >32/16] | 50/0/50 | [8: 8] | 100/0/0 | 100/0/0 | [≤4: ≤4] | [≤4: ≤4] | [≤4: ≤4] |
| 1. *hydrophila* | 2 | 100/0/0 | 100/0/0 | [≤4: ≤4] | [≤1: ≤1] | [≤4: ≤4] | 100/0/0 | 100/0/0 | 50/0/50 | 100/0/0 | [>32: >32] | [>32/16: >32/16] | 50/50/0 | [>32: >32] | 100/0/0 | 100/0/0 | [≤4: ≤4] | [≤4: ≤4] | [≤4: ≤4] |
| 1. *dhakensis* | 3 | 100/0/0 | 100/0/0 | [≤4: ≤4] | [≤1: ≤1] | [≤4: ≤4] | 75/25/0 | 100/0/0 | 75/0/25 | 100/0/0 | [>32: >32] | [>32/16: >32/16] | 75/25/0 | [>32: >32] | 100/0/0 | 100/0/0 | [≤4: ≤4] | [≤4: ≤4] | [>32: >32] |
| 1. *media* | 2 | 100/0/0 | 100/0/0 | [≤4: ≤4] | [≤1: ≤1] | [≤4: ≤4] | 100/0/0 | 100/0/0 | 100/0/0 | 100/0/0 | [>32: >32] | [>32/16: >32/16] | 100/0/0 | [>32: >32] | 100/0/0 | 100/0/0 | [≤4: ≤4] | [≤4: ≤4] | [≤4: ≤4] |

**Table S1.** Antimicrobial susceptibility profiles of *Aeromonas* species. AMK, amikacin, AXO, ceftriaxone TOB, tobramycin, MIN, minocycline, SXT, trimethoprim/sulfamethoxazole, CIP, ciprofloxacin
